# Genome-Wide Scan for Runs of Homozygosity Identifies Candidate Genes in Three Pig Breeds

**DOI:** 10.3390/ani9080518

**Published:** 2019-08-01

**Authors:** Rui Xie, Liangyu Shi, Jiaxin Liu, Tianyu Deng, Lixian Wang, Yang Liu, Fuping Zhao

**Affiliations:** 1 Department of Animal Genetics, Breeding and Reproduction, National Experimental Teaching Demonstration Center of Animal Science, College of Animal Science and Technology, Nanjing Agricultural University, Nanjing 210095, China; 2 Key Laboratory of Animal Genetics, Breeding and Reproduction (Poultry) of Ministry of Agriculture, Institute of Animal Science, Chinese Academy of Agricultural Sciences, Beijing 100193, China

**Keywords:** runs of homozygosity, inbreeding coefficient, pig, candidate genes

## Abstract

**Simple Summary:**

Runs of homozygosity (ROH) are the DNA segments that harbor uninterrupted stretches of homozygous genotype segments in the genome that are present in an individual due to the transmission of identical haplotypes from parents to their offspring. ROHs are widely used as predictors of whole-genome inbreeding levels in animals and identify highly selected genomic regions. In this study, we investigated the ROH distributions on the whole genome in three pig populations (Landrace, Songliao black and Yorkshire pigs). Moreover, inbreeding coefficients based on ROH were calculated and genes were annotated in the genomic regions with a high frequency of ROH. Results showed that Songliao black pigs had higher inbreeding in recent generations and ten genes related to economically important traits were located within ROH regions. Our findings provide a reference for developing breeding programs to maintain diversity and fitness in these breeds.

**Abstract:**

Runs of homozygosity (ROH) are contiguous homozygous genotype segments in the genome that are present in an individual since the identical haplotypes are inherited from each parent. The aim of this study was to investigate the frequency and distribution of ROH in the genomes of Landrace, Songliao black and Yorkshire pigs. We calculated two types of genome inbreeding coefficients and their correlation, including the inbreeding coefficient based on ROH (*F_ROH_*) and the inbreeding coefficient based on the difference between the observed and expected number of homozygous genotypes (*F_HOM_*). Furthermore, we identified candidate genes in the genomic region most associated with ROH. We identified 21,312 ROH in total. The average number of ROH per individual was 32.99 ± 0.38 and the average length of ROH was 6.40 ± 0.070 *Mb* in the three breeds. The *F_ROH_* results showed that Yorkshire pigs exhibited the highest level of inbreeding (0.092 ± 0.0015) and that Landrace pigs exhibited the lowest level of inbreeding (0.073 ± 0.0047). The average correlation between *F_ROH_* and *F_HOM_* was high (0.94) within three breeds. The length of ROH provides insight into the inbreeding history of these three pig breeds. In this study, Songliao black pigs presented a higher frequency and average length of long ROH (>40 *Mb*) compared with those of Landrace and Yorkshire pigs, which indicated greater inbreeding in recent times. Genes related to reproductive traits (*GATM*, *SPATA46*, *HSD17B7*, *VANGL2*, *DAXX*, *CPEB1*), meat quality traits (*NR1I3*, *APOA2*, *USF1*) and energy conversion (*NDUFS2*) were identified within genomic regions with a high frequency of ROH. These genes could be used as target genes for further marker-assisted selection and genome selection.

## 1. Introduction

Runs of homozygosity (ROH) were first introduced by J Gibson et al. [1], who defined the ROH as contiguous homozygous genotype segments in the genome that are present in an individual due to the transmission of identical haplotypes from parents to their offspring. Studies have found that long homozygous fragments in the genome are widespread in all populations [1,2,3,4,5]. Inbreeding is the mating of relatives, which can increase homozygosity in a population, and with an increase in the inbreeding level, the probability of homozygosity of harmful recessive genes also increases, which can lead to decreases in the fecundity, viability and phenotype of offspring, which is referred to as inbreeding depression [6]. There are several factors influencing the generation of ROH, such as inbreeding, natural and artificial selection, genetic drift and population bottlenecks [7,8]; however, inbreeding is considered the most important factor [9,10,11]. In the process of evolution and the development of variety under the influence of natural selection and manual selection, different mating systems, different selection directions, different population sizes and different population development histories will form a unique ROH distribution pattern in the animal genome; therefore, the number, length, distribution and frequency of ROH in animal genomes provide rich genetic background information, such as animal population histories and inbreeding levels [12,13,14].

The inbreeding coefficient (*F*) is generally used to evaluate the extent of an individual’s inbreeding. Traditionally, the calculation of the inbreeding coefficient is based on pedigree information (*F_ped_*). However, this method has some shortcomings: (i) Meiosis is stochastic, with random variations occurring in the process of obtaining genetic material from both parents in offspring and these variations increase with an increase in the number of meioses. (ii) The inbreeding coefficient of a pedigree, which is the expected value of identity by descent (IBD) probability [15] is relative to that of base group, which assumes that no individuals exhibit inbreeding; however, pedigree information can only be recorded for limited generations, and historical inbreeding is ignored, leading true homozygosity to be underestimated [16,17,18,19]. (iii) The accuracy of *F_ped_* depends on the integrity and accuracy of pedigree information. In animal production records, pedigree errors often occur during mating, calving or pedigree entry. The average pedigree error rate of dairy cows worldwide is approximately 11% [20].

The advent of high-throughput genotyping technology has provided new methods for the assessment of inbreeding based on single nucleotide polymorphisms (SNPs), such as the evaluation of genome inbreeding based on the proportion of runs of homozygosity (ROH) as an alternative to traditional pedigree-based inbreeding coefficients. Gomez-Raya et al. [21] used simulated genetic data and real genetic data to compare four methods: (i) *F_PED_*: *F* based on pedigree information; (ii) *F_h_*: *F* based on excess SNP homozygosity; (iii) *F_alt_*: An alternative estimate of *F* predicted to show lower error; and (iv) *F_ROH_*: *F* based on runs of homozygosity for estimating inbreeding coefficients, and they found that *F_ROH_* was the most powerful method for detecting inbreeding effects among the four methods. Mehrnush et al. [22] compared the inbreeding coefficient based on the pedigree (*F_PED_*), the genome relational matrix inbreeding coefficient (*F_GRM_*), the inbreeding coefficient based on ROH (*F_ROH_*) and the true inbreeding coefficient (*F_TRUE_*) based on the North American Holstein dairy cattle population and found that *F_ROH_* was closest to the true inbreeding coefficient. ROH are widely used to predict the whole genome inbreeding levels of individuals and populations [23].

The selection of animals according to a specific production direction will increase the homozygosity of the selected regions of the genome, which leads to the occurrence of ROH [24]. As a result, genomic regions with a high frequency of ROH can be used to detect associations between genes and economic traits of interest [25]. Zhang et al. [26] identified genes related to growth rates and immunity in western pig breeds and genes related to reproductive traits, adaptive traits and meat traits in Chinese pig breeds in short genomic regions with a high frequency of ROH, and the candidate gene PRM1, which is associated with high fecundity of Chinese pig breeds, was identified in long genomic regions with a high frequency of ROH.

In this study, we used the Illumina porcine 60 K SNP BeadChip to characterize Landrace, Songliao black and Yorkshire pigs. Based on ROH, we calculated the genomic inbreeding coefficient (*F_ROH_*) and identified candidate genes in genomic regions with a high frequency of ROH. The results provide unique insight into the population history and structure of the three pig breeds.

## 2. Materials and Methods

### 2.1. Ethics Statement

All experimental procedures used in this study were carried out in accordance with the guidelines for the care and use of laboratory animals of the Institutional Animal Care and Use Committee of the College of Animal Science and Technology, Nanjing Agricultural University (No. SYXK(SU)2017-0027) and all methods involving pigs were in accordance with the Standards for the Administration of Experimental Practices (Jiangsu, China).

### 2.2. SNP Genotyping and Quality Control

A total of 646 individuals from three pig breeds were included in our study: 83 Landrace, 86 Songliao black and 477 Yorkshire pigs. Genomic DNA was extracted from ear tissue and genotyped with the Illumina porcine 60 K SNP BeadChip (Illumina, San Diego, CA, USA). We only focused on autosomal SNPs for further analyses. The software PLINK (v1.90) [27] was used for quality control of the data and the following standards were set: (i) Removal of SNP loci with a call rate of less than 0.95 and unknown positions; (ii) Removal of SNP loci with a minor allele frequency (MAF) of less than 0.05; and (iii) Discarding of individuals with a call rate of less than 0.90. SNP genome coordinates were obtained from the *Sus scrofa* 10.2 porcine genome reference assembly.

### 2.3. Runs of Homozygosity Detection

ROH were detected with the detectRUNS package of R software [27]; we defined ROH according to the following criteria: (i) The minimum number of SNPs in a sliding window was 50; (ii) One heterozygous genotype and no more than two missing SNPs were allowed per window; (iii) The minimum ROH length was set to 1 *Mb* to eliminate the impact of strong linkage disequilibrium (LD); (iv) The minimum SNP density was 1 SNP every 500 kb and the maximum gap between consecutive SNPs was set to 1 *Mb* to avoid affecting the length of ROH with a low SNP density; and (v) To minimize the number of the false-positive ROH, the minimum number of SNPs that constituted the ROH (*l*) was calculated with the method proposed by Lencz et al. [28], $l=\frac{\ln\alpha /(n_{s}\times n_{i})}{\ln(1-het)}$, where α is the percentage of false-positive ROH (set to 0.05 in the present study), *n_s_* is the number of SNPs per individual, *n_i_* is the number of individuals and *het* is the proportion of heterozygosity across all SNPs. After calculation, the minimum number of SNPs constituting an ROH was set to 43.

In this study, we classified ROH into five different categories according to their physical length: 1 to <5 *Mb*, 5 to <10 *Mb*, 10 to <20 *Mb*, 20 to <40 *Mb* and >40 *Mb*. For each length category, we computed the frequency of ROH numbers and the average length of an ROH per breed.

### 2.4. Inbreeding Coefficient

To verify the accuracy of *F_ROH_*, we evaluated the genomic coefficient via two methods. (1) *PLINK* was used to measure the inbreeding coefficient based on the difference between the observed and expected numbers of homozygous genotypes (*F_HOM_*) [27]. The calculation formula was as follows: $F_{HOM}=\left(E_{HOM}-O_{HOM}\right)/\left(L-E_{HOM}\right)$, where *L* is the number of genotyped autosomal SNPs, *E_HOM_* is the number of expected homozygous genotypes and *O_HOM_* is the number of observed homozygous genotypes. (2) The inbreeding coefficient based on the proportion of autosomes covered in runs of homozygosity per individual (*F_ROH_*) was determined. *F_ROH_* was calculated as follows: $F_{ROH}=L_{ROH}/L_{AUTO}$, where *L_ROH_* is the total length of ROH on autosomes and *L_AUTO_* is the total length of the autosomes covered by SNPs, which was 2450.71 *Mb*. Furthermore, the correlation between *F_ROH_* and *F_HOM_* was calculated for the three breeds.

### 2.5. Detection of Common Runs of Homozygosity

To identify genomic regions with a high frequency of ROH, we calculated the percentage of the occurrence of SNPs in ROH by counting the number of times a SNP was detected in a particular ROH across individuals and selected the SNP regions showing a percentage higher than 40% as genomic regions with a high frequency of ROH for subsequent analyses. We used the porcine reference genome annotation file from the Ensemble database to annotate the genes identified at particular genome coordinates for all selected regions (ftp://ftp.ensembl.org/pub/release-89/gtf/sus_scrofa/Sus_scrofa.Sscrofa10.2.89.gtf.gz); the function of these genes was annotated at the NCBI website (https://www.ncbi.nlm.nih.gov/); moreover, we conducted an extensive literature search.

## 3. Results

### 3.1. Distribution of Runs of Homozygosity

After filtering, 37,540, 36,476 and 36,180 SNPs and 83, 86 and 477 individuals were retained from the Landrace, Songliao and Yorkshire pigs, respectively. To better analyze the ROH results of the three breeds, the 30,282 common SNPs were retained for subsequent ROH analysis.

Among the 646 individuals, 644 (99.6%) exhibited at least one ROH longer than 1 *Mb*, whereas no ROH were identified in two individuals, including one individual in the Yorkshire population and one individual in the Songliao population. A total of 21,312 ROH were identified in 644 individuals. Among all identified ROH, the lengths of 12,192 ROH were shorter than 5 *Mb*, while those of 6077 ROH ranged from 5 to 10 *Mb*, those of 2213 ROH from 10 to 20 *Mb*, those of 635 ROH from 20 to 40 *Mb* and 195 ROH were longer than 40 *Mb* (see Table 1).

Table 2 summarizes the average number and length of ROH in the three breeds. As shown in Table 2, the average number of ROH per individual was 32.99 ± 0.38 and the average length of ROH was 6.40 ± 0.070 *Mb* in the investigated individuals. Among all ROH, the longest ROH was 126.75 *Mb*, which consisted of 2318 SNPs and occurred on chromosome 14 in the Yorkshire pig population. The individual in which the largest number of ROH (59 ROH) was detected and the individual in which the lowest number of ROH (4 ROH) was detected were in the Yorkshire pig population, despite the existence of individuals in which no ROH were detected. The longest average ROH length among the three breeds was found in the Songliao black pig population (7.49 ± 0.31 *Mb*); the lowest average ROH length among the three breeds was found in the Yorkshire pig population (6.21 ± 0.063 *Mb*).

**Figure 1:**
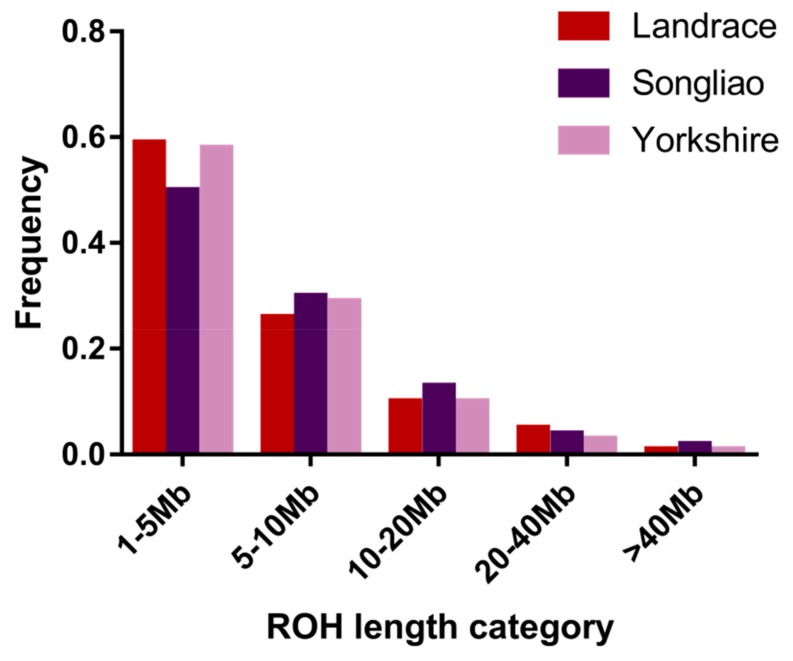
Classification of runs of homozygosity (ROH) in five different length categories (from 1–5 *Mb* to more than 40 *Mb*) (x-axis) and the frequency of ROH in each category in each breed (y-axis).

**Figure 2:**
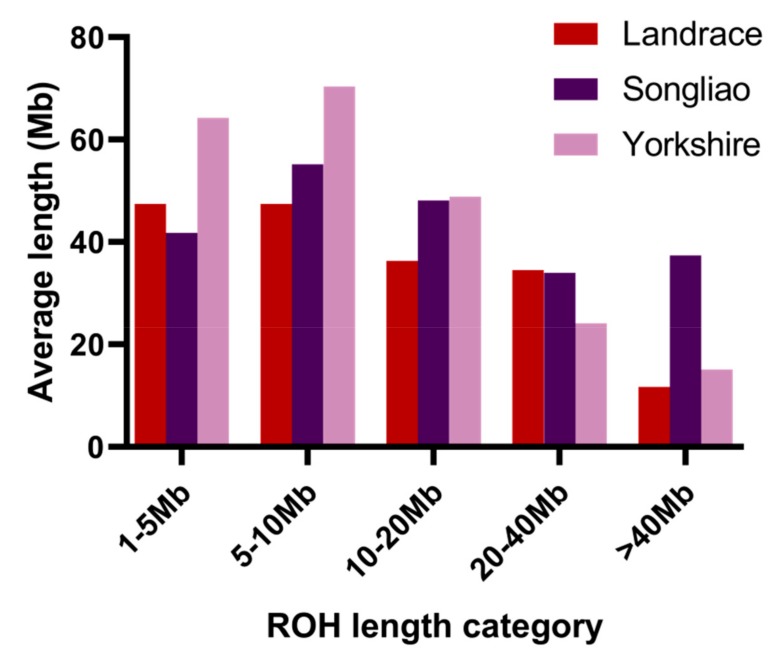
Classification of runs of homozygosity (ROH) in five different length categories (from 1–5 *Mb* to more than 40 *Mb*) (x-axis) and average length of runs of homozygosity (ROH) in five different length categories (y-axis).

The frequency of ROH numbers within the five categories of ROH length (1–5 *Mb*, 5–10 *Mb*, 10–20 *Mb*, 20–40 *Mb* and >40 *Mb*) is illustrated (see Figure 1). The length of ROH mainly fell within 1–10 *Mb* and the number of ROH within 1–10 *Mb* accounted for 85.72% of the total number of ROH. In the 1–5 *Mb* category, Songliao black pig exhibited a lower frequency of ROH than the Landrace and Yorkshire pigs (*p* < 0.001) but the highest frequency of ROH was found in the Songliao black pig population in the >40 *Mb* category (*p* < 0.001). Furthermore, the average length of ROH per breed within each ROH length category is shown in Figure 2.

In the 1–5 *Mb* category, the Yorkshire pigs presented the greatest average ROH length (64.25 *Mb*) among the three breeds and Songliao black pigs presented the shortest average ROH length (41.79 *Mb*) among the three breeds; in the >40 *Mb* category, Songliao black pigs exhibited a greater average ROH length (37.34 *Mb*) compared with Landrace and Yorkshire pigs, while Landrace pigs presented the shortest average ROH length (11.69 *Mb*) among the three breeds.

**Figure 3:**
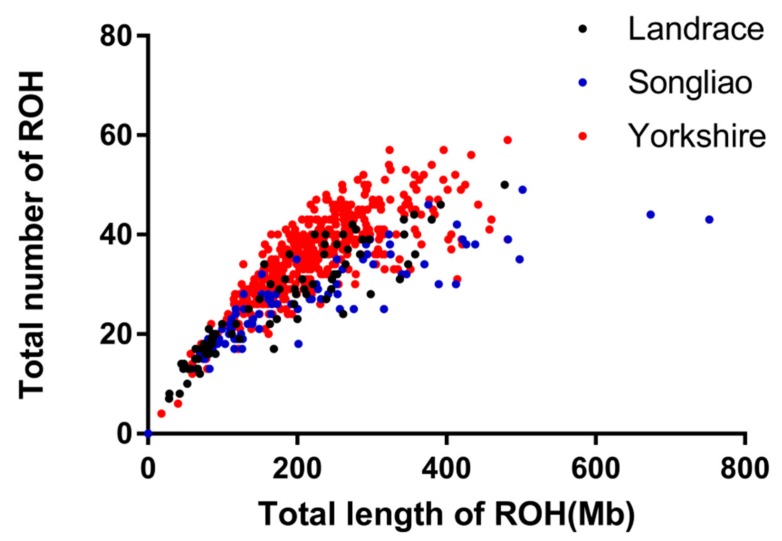
Total genomic length (*Mb*) covered by ROH per individual (x-axis) and total number of ROH per individual (y-axis).

The relationship between the total genomic length covered by ROH per individual and the total number of ROH per individual is plotted in Figure 3. Yorkshire pigs exhibited a larger number of ROH than Songliao black pigs and Landrace pigs and Songliao black pigs presented some extreme individuals with a length of ROH that covered more than 500 *Mb*.

### 3.2. Inbreeding Coefficient of Runs of Homozygosity (F_ROH_)

**Figure 4:**
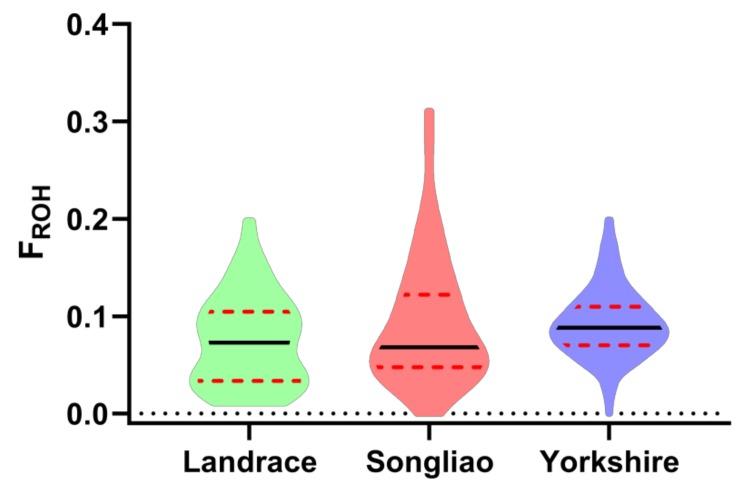
Distribution of runs of homozygosity inbreeding coefficients within each breed.

The average inbreeding coefficient, its range of variation in the three pig breeds and its distribution are summarized in Table 2. The average *F_ROH_* of Yorkshire pigs was highest among these three populations, at 0.092. The average *F_ROH_* of Landrace pigs (0.073) was lowest. The average *F_ROH_* of Songliao black pigs was between those of the Landrace pigs and Yorkshire pigs. The *F_HOM_* results indicated similar conclusions. At the individual level, the individuals with the highest *F_ROH_* appeared in the Songliao black pigs (0.31) and Songliao black pigs exhibited more individuals with extreme values compared with other populations (see Figure 4); when individuals without any identified ROH were considered, the individuals with the lowest *F_ROH_* appeared in the Yorkshire pig population (0.0075). The individuals with the highest *F_HOM_* occurred in the Songliao black pig population and the individuals with the lowest *F_HOM_* also occurred in the Yorkshire pig population. The correlations between *F_ROH_* and *F_HOM_* were 0.95, 0.98 and 0.93 in Landrace, Songliao black and Yorkshire, respectively. The average correlation between *F_ROH_* and *F_HOM_* in the three breeds was 0.94.

We also summarized the percentage of chromosome coverage by ROH of each length class in each breed in Table 3. The highest chromosome coverage by ROH was on chromosome 14 in the Landrace and Songliao breeds and on chromosome 4 in the Yorkshires, while the lowest chromosome coverage by ROH was on chromosome 12 in Landrace and Yorkshires and on chromosome 6 in the Songliao black breed.

### 3.3. Genomic Regions with a High Frequency of ROH

**Figure 5:**
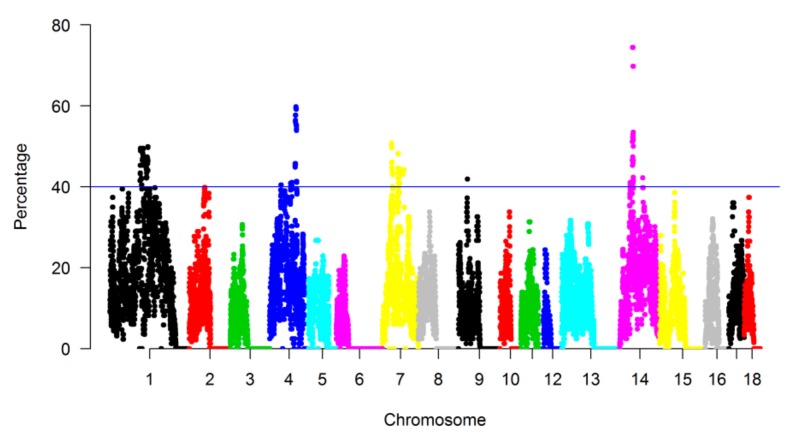
Manhattan plot of the occurrence (%) of single nucleotide polymorphisms (SNPs) in ROH across individuals. A colorful dot stand for a SNP.

The genomic regions that were most commonly associated with ROH were identified in the three pig breeds, and we assessed the proportion of SNPs in ROH by calculating the frequency of SNPs occurring in those ROH across all individuals. The result was plotted against the position of the SNP along the chromosome (Figure 5). A total of 22 regions were detected as genomic regions with a high frequency of ROH, among which 4 genomic regions did not harbor any genes, and a total of 289 genes were identified in the remaining 18 genomic regions (see Supplementary Materials Table S1).

## 4. Discussion

The frequency and distribution of ROH in the genome of three pig breeds (Landrace, Songliao black and Yorkshire) were analyzed with the Illumina porcine 60 K SNP BeadChip. The abundance, length and genomic distribution of ROH constitute a valuable source of information about the demographic history of livestock species [13]. Long ROH can indicate the kinship of recent generations because the shorter the generations, the less likely an ROH fragment will be interrupted by reorganization and longer ROH indicate a higher probability of inbreeding in the population [29,30]. Our results showed significant differences in the number and length of ROH within the three breeds. The ROH numbers and lengths of Landrace pigs and Yorkshire pigs were shorter than those of Songliao black pigs in the >40 *Mb* category, which indicated greater inbreeding in Songliao black pigs in recent times. This result was consistent with previous research by [26]. Landrace and Yorkshire pigs are western commercial breeds with a long history of cultivation but Songliao black pigs are a Chinese breed; by the end of 2008, the number of boars in the core population of Songliao black pigs was only 160 [31]. Hence, inbreeding was unavoidable because of the limited number of boars.

Animals with the same cumulative length of ROH presented different numbers of ROH with different lengths because of their different distances from the last common ancestor [9]. As shown in Figure 3, the total genomic length (*Mb*) covered by ROH per individual was approximately proportional to the total number of ROH per individual; with an increase in the total length of individual ROH, the total number of ROH increases synchronously. To some extent, these results could reflect the inbreeding level or the differences in population history among different populations, where the higher the inbreeding level, the greater the number of ROH in the genome and the longer the total length of ROH. Some extreme individuals with ROH lengths exceeding 500 *Mb* were identified among the Songliao black pigs and the longest total ROH length in an individual was 752.25 *Mb*. This result reflected the lack of effective management of inbreeding in the Songliao black pig population. Similar results regarding the ROH distribution between length and number have been reported in cattle [4,32], pigs [33] and sheep [9]. Landrace and Yorkshire are western commercial breeds that have been subjected to systematic breeding; Songliao black pigs are a local Chinese breed that has not been subjected to systematic breeding. Generally, the *F_ROH_* of Songliao black pigs should be smaller than those of Landrace and Yorkshire pigs but the results of our study indicated that the *F_ROH_* of Songliao black pigs was between those of Landrace pigs and Yorkshire pigs. This result may be due to the small effective population size, such that the small effective population may have resulted in a high degree of inbreeding in recent generations [7]. The inbreeding coefficient of the Landrace pigs calculated as *F_HOM_* was negative, which may have been due to the small number of Landrace pigs in our study and random sampling errors could also lead to a negative result [27].

In this study, we identified a total of 10 genes reported to be associated with pig economic traits based on genomic regions with a high frequency of ROH (see Table 4). In the Yorkshire pigs, the *GATM* gene was identified on chromosome 1 and reported to be associated with placental development in Yorkshire and Duroc pigs [34]. Three genes were identified within the ROH on chromosome 4: *SPATA46* is a gene, encoding a novel protein in mouse testis and deficiency of *SPATA46* can lead to subfertility in male mice [35]; the *HSD17B7* gene is expressed in porcine endometrium and oocytes and is related to reproductive traits [36,37]; the *VANGL2* gene is associated with embryo implantation in mice and is essential for embryonic development, cell adhesion, migration and polarity [38]. We also identified three genes associated with meat quality traits on chromosome 4: *NR1I3* has been reported as a candidate gene for promoting a reduction in backfat thickness and increasing lipid deposition capacity among pigs [39] and it has been reported to be associated with the feeding efficiency of Nellore cattle [40]; *APOA2* encodes a protein implicated in triglyceride, fatty acid and glucose metabolism and the *APOA2* gene is located within a reported quantitative trait locus (QTL) region for fatty acid composition traits, fatness and growth traits in pigs [41]; and the *USF1* gene has been found to play an important role in many meat production traits of pigs, such as average backfat thickness, loin eye width, lean meat percentage and loin eye height [42]. Furthermore, we identified an interesting gene on chromosome 4, *NDUFS2*, which has been reported to be associated with energy production and transformation in pigs and to show differential expression in pig skeletal muscle [43]. The *DAXX* gene, located on chromosome 7 of Yorkshire pigs, was recently reported to exhibit the highest expression in the middle stage of mouse testis development and may be involved in the regulation of spermatogenesis in mice [44]. We found a common gene on chromosome 7 of Songliao black and Landrace pigs: The *CPEB1* gene has been reported to participate in Cyclin B translation and meiotic resumption in porcine oocytes [45].

## 5. Conclusions

In this study, the existence and distribution of ROH in the three breeds (Landrace, Songliao and Yorkshire) were explored based on porcine 60 K SNP BeadChip data. Our study showed that Songliao black pigs exhibited a higher frequency and average length of long ROH (>40 *Mb*), indicating higher inbreeding in Songliao black pigs in recent times. Several genes related to reproductive traits (*GATM, SPATA46, HSD17B7, VANGL2, DAXX, CPEB1*), meat quality traits (*NR1I3, APOA2, USF1*) and energy conversion (*NDUFS2*) are located in genomic regions with a high frequency of ROH. These genes can be used as target genes for future marker-assisted selection.

## Figures and Tables

**Table 1:** Summary of the number of runs of homozygosity (ROH) in different categories in each breed.

	1–5 *Mb*	5–10 *Mb*	10–20 *Mb*	20–40 *Mb*	>40 *Mb*
Landrace	1269	564	216	104	16
Songliao	1152	676	306	101	53
Yorkshire	9771	4837	1691	430	126

**Table 2:** Descriptive statistics for runs of homozygosity and inbreeding coefficients (F) within each breed.

Breed	Sample Size	SNP Number *	Average Length(*Mb*)		Average Number		*F_ROH_*		*F_HOM_*	$r_{\left(F_{ROH},F_{HOM}\right)}$
			Mean ± SE	Range	Mean ± SE	Range	Mean ± SE	Range		
Landrace	83	44~1853	6.33 ± 0.21	28.11~478.17	26.13 ± 1.14	7~50	0.073 ± 0.0047	0.012~0.20	−0.0013 ± 0.0094	0.95
Songliao	86	46~2100	7.49 ± 0.31	0~752.25	26.60 ± 0.96	0~49	0.089 ± 0.0063	0~0.31	0.0050 ± 0.010	0.98
Yorkshire	477	43~2318	6.21 ± 0.063	0~482.18	35.34 ± 0.39	0~59	0.092 ± 0.0015	0~0.20	0.034 ± 0.0031	0.93
Average	215.33	43~2318	6.40 ± 0.070	0~752.25	32.99 ± 0.38	0~59	0.089 ± 0.0015	0~0.31	0.026 ± 0.0031	0.94

*: the range of single nucleotide polymorphisms (SNPs) involved in ROH; $r_{\left(F_{ROH},F_{HOM}\right)}$, correlation between *F_ROH_* and *F_HOM_*.

**Table 3:** The percentage of chromosome coverage (%) by ROH of each length classes in each breed.

CHR	Length (*Mb*) *	Landrace						SongLiao						Yorkshire					
		1–5 *Mb*	5–10 *Mb*	10–20 *Mb*	20–40 *Mb*	>40 *Mb*	Total	1–5 *Mb*	5–10 *Mb*	10–20 *Mb*	20–40 *Mb*	>40 *Mb*	Total	1–5 *Mb*	5–10 *Mb*	10–20 *Mb*	20–40 *Mb*	>40 *Mb*	Total
1	315.29	1.35	2.85	2.23	3.75	2.06	**12.24**	1.25	3.27	3.08	2.24	1.75	**11.59**	2.47	5.01	4.43	3.36	1.74	**17.01**
2	162.55	1.73	2.73	1.44	0.56	0	**6.46**	1.69	2.07	1.40	0.71	1.02	**6.89**	2.07	1.99	1.23	0.31	0.32	**5.92**
3	144.77	1.93	1.22	0.39	0	0	**3.54**	1.54	1.25	0.64	0.23	0.46	**4.12**	2.24	1.38	0.57	0.20	0.14	**4.53**
4	143.44	3.47	2.44	0.88	2.98	0	**9.77**	2.53	4.31	3.89	3.55	1.56	**15.84**	4.25	7.90	3.97	1.50	0.98	**18.60**
5	111.49	1.42	1.42	3.33	0.58	0	**6.75**	1.47	3.78	2.25	0.28	0	**7.78**	2.86	2.26	1.02	0.05	0	**6.19**
6	157.75	1.73	0.60	0.09	0	0	**2.42**	0.78	0.66	0.22	0	0	**1.66**	1.63	0.49	0.20	0.09	0.06	**2.47**
7	134.75	2.69	3.63	4.62	1.71	0	**12.65**	3.56	5.33	5.76	1.74	0.70	**17.09**	3.95	3.38	3.79	0.89	0.13	**12.14**
8	148.48	0.97	1.43	1.50	2.01	0	**5.91**	1.08	0.82	0.35	1.57	0.75	**4.57**	1.75	1.91	0.96	0.56	0.51	**5.69**
9	153.65	1.81	1.24	0.58	0.22	0	**3.85**	2.62	2.87	1.28	0.16	0.80	**7.73**	2.59	1.69	0.64	0.28	0.20	**5.40**
10	79.38	2.89	1.31	1.30	0.40	0	**5.90**	1.94	1.92	1.44	0.98	0	**6.28**	2.88	0.86	0.70	0.28	0	**4.72**
11	87.68	2.70	1.14	0.34	0.75	0	**4.93**	0.70	1.71	1.41	3.18	2.08	**9.08**	2.19	2.53	2.45	0.20	0.72	**8.09**
12	63.58	1.03	0.45	0.43	0	0	**1.91**	2.26	1.65	0.78	0	0	**4.69**	1.42	0.42	0.25	0.07	0	**2.16**
13	218.61	1.20	1.25	0.67	0.66	0.82	**4.60**	1.33	1.37	1.05	1.23	2.31	**7.29**	1.90	2.61	1.85	0.95	0.47	**7.78**
14	153.83	3.24	4.07	2.47	2.69	2.21	**14.68**	2.59	1.84	4.00	2.96	6.79	**18.18**	4.36	4.86	3.79	2.11	2.56	**17.68**
15	157.67	1.98	1.60	1.49	0.74	0	**5.81**	1.41	0.97	1.43	1.00	1.75	**6.56**	1.55	2.15	1.41	0.46	0.20	**5.77**
16	86.89	1.08	1.28	0.17	0	0	**2.53**	1.02	1.06	1.16	1.44	1.19	**5.87**	3.48	3.10	1.14	0.47	0.31	**8.50**
17	69.69	2.93	1.94	2.80	1.88	0	**9.55**	1.79	3.70	1.29	1.52	4.13	**12.43**	4.60	2.05	1.02	0.53	0	**8.20**
18	61.21	2.34	1.65	0.97	3.61	0	**8.57**	1.74	0.85	1.73	0.64	0	**4.96**	2.39	1.43	1.08	1.10	0	**6.00**

*: Chromosome length of *Sus scrofa* 10.2 porcine genome.

**Table 4:** Candidate genes located in genomic regions with a high frequency of ROH associated with pig economic traits.

Population	CHR	Start (bp)	End (bp)	Gene Symbol	Distance between ROH Region and Gene *	Function
Landrace	7	57617069	58768368	*CPEB1*	0.17 *Mb*	Reproduction
Songliao	7	57554035	58768368	*CPEB1*	0.24 *Mb*	Reproduction
Yorkshire	1	135628006	141358502	*GATM*	5.21 *Mb*	Reproduction
	4	94734328	100297082	*HSD17B7*	0.84 *Mb*	Reproduction
				*SPATA46*	1.17 *Mb*	Reproduction
				*VANGL2*	3.21 *Mb*	Reproduction
				*NDUFS2*	2.37 *Mb*	Energy conversion
				*NR1I3*	2.35 *Mb*	Meat quality
				*APOA2*	2.36 *Mb*	Meat quality
				*USF1*	2.64 *Mb*	Meat quality
	7	33743648	35729978	*DAXX*	0.44 *Mb*	Reproduction

* The distance between genes and ROH regions was calculated as follows: The starting coordinate of the gene minus the starting coordinate of the ROH region; all candidate genes are located in the ROH region.

## Supplementary Materials

The following are available online at https://www.mdpi.com/2076-2615/9/8/518/s1, Table S1: Candidate genes located in genomic regions with a high frequency of ROH.
