# Supplementary material for: Genome-Wide Scan for Runs of Homozygosity Identifies Candidate Genes in Three Pig Breeds
Source: Animals (Basel). 2019 Aug 1;9(8):518. doi: 10.3390/ani9080518 (PMC6720638; doi:10.3390/ani9080518)
Supplement: Supplementary file 1 [file animals-09-00518-s001.pdf]

Additional Table 1 **C**andidate genes located in genomic regions with a high frequency of ROH

Population	Chromosome	Position(Mb)	Number. of SNP	Gene name
Landrace	7	57.62~58.77	21	*RPS17,FURIN,HDDC3,RCCD1,WHAMM,FES, MAN2A2,UNC45A,UROC1,ZXDC,CPEB1,PDE8A,FSD2,SLC41A3*
	7	58.92~60.12	10	*ACAN,HAPLN3,FANCI,POLG,MFGE8,ABHD2, RHCG,RLBP1*
	7	62.40~64.65	17	*IMP3,NEIL1,COMMD4,C15orf39,RPP25,MPI, CPLX3,LMAN1L,CYP1A1,ISLR,C15orf59,SNUPN,MAN2C1,PPCDC,SCAMP5,COX5A,FAM219B,SCAMP2,ULK3,CYP1A2,CLK3,PML,STOML1, NPTN,HCN4,PTPN9,CSK,EDC3,SIN3A*
	14	47.60~48.67	29	*MN1,PITPNB,TTC28*
Songliao	7	57.55~58.77	24	*RPS17,FURIN,HDDC3,RCCD1,WHAMM,FES, MAN2A2,UNC45A,UROC1,ZXDC,HOMER2, CPEB1,PDE8A,FSD2,SLC41A3*
	7	71.28~80.08	91	*GPR33,GZMB,AP4S1,SCFD1,NOVA1,HEATR5A, HECTD1,G2E3,STXBP6*
	14	45.86~50.03	114	*CRYBB1,CRYBA4,ZNRF3,RHBDD3,GAS2L1, RASL10A,UQCR10,CRYBB3,CRYBB2,SEZ6L,HPS4,SRRD,TFIP11,TPST2,MN1,EMID1,EWSR1,AP1B1,NIPSNAP1,KIAA1671,MYO18B,ASPHD2, PITPNB,MTMR3,GRK3,TTC28,KREMEN1*
Yorkshire	1	112.05~118.03	79	*ONECUT2,SUMO1,POLI,C18orf54,RAB27B, CCDC68,TXNL1,FECH,NARS,MTFMT,SPG21, PIF1,MBD2,WDR7,ANKDD1A*
	1	120.25~124.07	63	*ANXA2,RORA,ICE2*
	1	135.63~141.36	64	*EID1,DUOXA2,COPS2,SECISBP2L,SLC12A1, MYEF2,SLC24A5,SEMA6D,SQRDL,GATM, C15orf48,SLC28A2,DUOX1,DUOXA1,DUOX2, TERB2,DTWD1,GALK2,SHC4,CEP152,FBN1, SLC30A4,SPATA5L1,SHF,SORD*

4	41.58~42.18	12	*RPL30,ERICH5,LAPTM4B,MTDH,POP1,RIDA*
4	77.27~78.13	8	*GGH*
4	79.61~81.08	10	*NSMAF,TOX*
4	82.39~82.61	9	*SDR16C5,PLAG1*
4	94.73~100.30	107	*RGS4,CCDC190,SPATA46,SH2D1B,FCRLB, CFAP126,MPZ,PCP4L1,NR1I3,APOA2,FCER1G ,PPOX,USP21,NIT1,TSTD1,USF1,NHLH1, PEA15,PIGM,KCNJ10,SLAMF9,TAGLN2,VSIG8 ,FCRL6,DUSP23,CRP,CRP,OR10J5, LOC100517891,OR10J3,OR10J1,OR6N1,OR6K6 ,OR6K3,OR6K2,OR10Z1,OR6P1,OR6Y1,CD1A, HSD17B7,UAP1,FCGR2B,TOMM40L,NDUFS2, B4GALT3,UFC1,DEDD,PFDN2,PVRL4,F11R, SLAMF7,LY9,CD244,CD48,CD84,SLAMF6, VANGL2,NCSTN,PEX19,CASQ1,ATP1A4,IGSF8, KCNJ9,CFAP45,FCER1A,CADM3,OR10K2, NUF2,RGS5,DDR2,SDHC,ADAMTS4, ARHGAP30,SLAMF1,COPA,ATP1A2,SLAMF8, ACKR1,OR6N2,OLFML2B*
7	33.74~35.73	38	*VPS52,B3GALT4,WDR46,PFDN6,TAPBP,ZBTB2 2,DAXX,BAK1,ZBTB9,PHF1,HMGA1,RPS18, RGL2, SYNGAP1,CUTA,UQCC2,LEMD2,MLN,SPDEF ,PACSIN1,SNRPC,TAF11,ITPR3,GRM4, UHRF1BP1,ANKS1A,DST*
7	37.13~37.98	12	*RAB44,PI16,PIM1,TMEM217,STK38,PPIL1, C6orf89,MTCH1,FGD2,RNF8,CPNE5*
14	47.26~49.72	61	*ZNRF3,RHBDD3,GAS2L1,RASL10A,MN1,EMID 1,EWSR1,AP1B1,NIPSNAP1,PITPNB,TTC28, KREMEN1*
